# Chromosome level assembly of the hybrid *Trypanosoma cruzi *genome

**DOI:** 10.1186/1471-2164-10-255

**Published:** 2009-06-01

**Authors:** D Brent Weatherly, Courtney Boehlke, Rick L Tarleton

**Affiliations:** 1 Center for Tropical and Emerging Global Diseases, University of Georgia, Athens, GA, USA

## Abstract

**Background:**

In contrast to the essentially fully assembled genome sequences of the kinetoplastid pathogens *Leishmania major *and *Trypanosoma brucei *the assembly of the *Trypanosoma cruzi *genome has been hindered by its repetitive nature and the fact that the reference strain (CL Brener) is a hybrid of two distinct lineages. In this work, the majority of the contigs and scaffolds were assembled into pairs of homologous chromosomes based on predicted parental haplotype, inference from TriTryp synteny maps and the use of end sequences from *T. cruzi *BAC libraries.

**Results:**

Ultimately, 41 pairs of chromosomes were assembled using this approach, a number in agreement with the predicted number of *T. cruzi *chromosomes based upon pulse field gel analysis, with over 90% (21133 of 23216) of the genes annotated in the genome represented. The approach was substantiated through the use of Southern blot analysis to confirm the mapping of BAC clones using as probes the genes they are predicted to contain, and each chromosome construction was visually validated to ensure sufficient evidence was present to support the organization. While many members of large gene families are incorporated into the chromosome assemblies, the majority of genes excluded from the chromosomes belong to gene families, as these genes are frequently impossible to accurately position.

**Conclusion:**

Now assembled, these chromosomes bring *T. cruzi *to the same level of organization as its kinetoplastid relatives and have been used as the basis for the *T. cruzi *genome in TriTrypDB, a trypanosome database of EuPathDB. In addition, they will provide the foundation for analyses such as reverse genetics, where the location of genes and their alleles and/or paralogues is necessary and comparative genome hybridization analyses (CGH), where a chromosome-level view of the genome is ideal.

## Background

The publication of the three trypanosomatid, or TriTryp, genomes in 2005 was an important advance in our understanding of the related parasites *Trypanosoma cruzi*, *Leishmania major*, and *Trypanosoma brucei*. However, as with other genomes [1,2] a published genome is not necessarily a completed genome. The issue of incompleteness within the TriTryp genomes is most striking with respect to *T. cruzi *which, because of its highly repetitive nature and the fact that the reference strain (CL Brener) is a hybrid of

two distinct *T. cruzi *lineages, resulted in the publication of 32,746 contigs only partially assembled into 638 scaffolds with no complete chromosomes [3]. Though this resulted in a genome suitable for identifying and studying individual genes, the absence of a chromosome-based assembly hinders analyses where the location of genes and their alleles and/or paralogues is important (e.g. comparative genome analysis and strategies for gene knockouts).

Fortunately, the genomes of *T. brucei *and *L. major *fared much better, providing not only nearly complete chromosomes but also synteny maps linking the majority of the core (and conserved) genes of the *T. cruzi *genome to *T. brucei *and *L. major *chromosomes [4-6]. In this study, we sought to organize the majority of the 32,746 *T. cruzi *contigs into chromosomes-size assemblies using a combination of scaffolds, synteny maps, and end sequences from *T. cruzi *BAC libraries. To support the soundness of the process, a set of chromosomes was validated experimentally via Southern blot analysis using individual genes as probes to confirm the predicted organization of each chromosome.

## Methods

### Genome Data

From version 5 of the *T. cruzi *genome [3] the following data were obtained: 32,746 contigs and 638 scaffolds, as well as the coordinates and annotation data of predicted genes, from the National Center for Biotechnology Information (NCBI) [7], results of TRIBE-MCL analysis to determine TriTryp clusters of orthologous genes (COGs) from published supplemental materials [3], and 1,131,562 whole genome shotgun sequence (wgs) reads from the Institute for Genome Research (TIGR). Note that the annotation data includes the predicted haplotype (i.e. "Esmeraldo-like" or "non-Esmeraldo-like") assignment for each gene. Additionally, 39,605 sequenced ends of the CHORI-105 BAC library (produced by Pieter de Jong at the Children's Hospital Oakland Research Institute and sequenced by TIGR), consisting of the TARBAC (avg. length 75 kb) and EPIFOS (avg. length 35 kb) libraries, were obtained from the GSS database at NCBI. All data were loaded into a generic feature format (GFF) database and visualized with the Generic Genome Browser (GBrowse) [8]. Telomeric repeats were identified by searching contigs for at least 9 contiguous runs of the hexameric repeat 5'-TTAGGG-3' [9].

### Synteny Maps

*T. brucei *chromosomes were used as the basis for the organization of the initial constructs of *T. cruzi *chromosomes. TriTryp synteny maps were downloaded from GeneDB [10] and were parsed using custom PERL scripts to determine an initial assignment of *T. cruzi *contigs and scaffolds based on the order of *T. cruzi *genes in the synteny map of each chromosome. Because the synteny maps are based on the order of genes on the *T. brucei *chromosomes, *T. cruzi *genes from the same contig (and thus the same region on a chromosome) may map to *T. brucei *genes on different chromosomes. To address this, each contig/scaffold was initially assigned to the construct that accounted for the greatest number of genes in the corresponding *T. brucei *chromosomes. Where available, the haplotype assignments for genes as previously annotated [3] were used to further designate Esmeraldo- and non-Esmeraldo-like homologous chromosomes. For situations where the haplotype was unknown or a sequenced contig was the result of the merging of sequence reads from both haplotypes, an assignment was made to the homologous chromosome accounting for the greatest number of genes on the contig or scaffold.

### Mapping of BAC Clones

The sequenced ends of the BAC clones from the CHORI105 library [11] were mapped to the contigs based upon BLAST [12] analysis. The mappings were classified as "high-confidence" if they matched no more than 2 contigs with 3 or fewer base mismatches but otherwise were considered "low-confidence". In general, the high-confidence mappings were used for the basis of organization, while the low-confidence mappings provided additional support. PERL scripts were then used to identify BAC clones for which both ends mapped to the same construct, ensuring that the ends were oriented properly to represent the clone (i.e. 1 sense and 1 anti-sense) and that the distance between the mapped ends was appropriate based on the average clone size of the library (TARBAC: avg. length 75 kb, EPIFOS: avg. length 35 kb). Using an iterative process of GBrowse visualization of each construct followed by contig/scaffold rearrangement to place syntenous sequences on the same chromosome and/or to correct the orientation of the sequenced BAC ends of the CHORI-105 BAC library, the constructs were split and/or expanded to generate the chromosomes.

To simplify the chromosome assembly process, only those BAC clones that spanned scaffolds were utilized, under the assumption that the organization of the previously assembled scaffolds [3] is correct. However, in some instances, scaffolds were found to contain both Esmeraldo- and non-Esmeraldo-like contigs. In these cases the scaffolds were split in order to place the contigs on the appropriate homologous chromosome.

Contigs/scaffolds containing genes absent in the *T. brucei *genome, and thus not fully represented in the first-pass constructs based on *T. cruzi/T. brucei *synteny, were also placed as dictated by the mapping of BAC end sequences.

### Model Chromosomes

To arrive at a single chromosome model for the CL Brener genome, consensus versions of each homologous chromosome pair were constructed by merging the gene features from the aligned contigs from both the Esmeraldo- and non-Esmeraldo-like haplotypes (one representative of each allele). While choosing a representative gene for loci containing 2 fully sequenced alleles is straightforward, in many cases 1 or both alleles were not fully sequenced and thus the genes exist in pieces on separate contigs (i.e. no contiguous sequence for the complete gene). In these cases, the most likely coordinates for the full-length locus was inferred.

Using SynView [13] for GBrowse, synteny maps were constructed using the model *T. cruzi *chromosomes as reference to compare to *T. brucei *and *L. major*. These *T. cruzi-*based TryTryp synteny maps were used to further validate the chromosome assemblies (see below).

### Dot Blot Validation

To validate the organization of the chromosomes, Southern "dot" blots were performed on overlapping BAC clones from the CHORI-105 library using genes along the chromosomes as probes. DNAs isolated from the CHORI-105 BAC library were used as targets, and gene-specific DNAs, either from a library of cloned genes in the Gateway pDONR201 plasmid [14] (Invitrogen) or produced by PCR amplification with gene specific primers, were used as probes in this process. Cloned genes were digested from pDONR plasmids with either *BsrGI *or *PsiI *and then labeled with Digoxigenin-11-dUTP using Roche's DIG-High Prime DNA labeling Kit and Detection Starter Kit as per manufacturer's instructions. Denatured target (BAC clone) DNA was applied to positively charged nylon membranes (Roche) and incubated with denatured, labeled probes in pre-heated hybridization buffer (DIG Easy Hyb Roche) at a hybridization temperature calculated using the Wahl formula [15] for 16 hrs. Membranes were then washed and spots were colormetrically detected according to the DIG High Prime DNA Labeling and Detection Starter kit I (Roche) as per manufacturer's instructions.

## Results

The goal of this study was to extend and improve the organization of the existing genome data for *T. cruzi *in order to construct a model of the core regions of the *T. cruzi *chromosomes. Accordingly, certain assumptions and design decisions were made to simplify the process. First, the organization of previously published scaffolds was assumed correct unless significant evidence suggested otherwise. This assumption is particularly important for the scaffolds rich in members of large gene families, as these scaffolds were included where possible but could not be validated further. Second, although some contigs are clearly the result of merging of sequences from both haplotypes, no global attempt was made to split misassembled contigs. Third, because many BAC ends mapped to multiple locations on the chromosomes or had mates that mapped to different chromosomes, completely disambiguating the organization of the contigs/scaffolds based on mapping of BAC ends was not possible, and thus placement decisions were made based on majority evidence. Finally, while some *T. cruzi *chromosomes are known to be triploid [16] the focus of this work was to differentiate the homologous chromosomes of the two parental haplotypes only, and thus all assemblies are presented as diploid. Where appropriate, we address consequences of these decisions.

### Chromosome Construction

TriTryp synteny maps for the *T. brucei *chromosomes were used as an initial platform on which to tentatively assemble 11 pairs of homologous "*T. brucei*-like" chromosomes from the *T. cruzi *genes, contigs, and scaffolds. Contigs that were not present in the *T. brucei *synteny maps but whose location in the *T. cruzi *constructs could be inferred were also added. These constructs were then systematically reorganized into *T. cruzi *chromosome-size pieces based on the preferential mapping of both ends of BAC clones that would link contigs or scaffolds in the proper orientation ("closed" BAC clones). Ultimately, 41 chromosomes were assembled using this approach, a number in agreement with the predicted number of *T. cruzi *chromosomes based upon pulsed-field gel electrophoresis (PFGE) [17-22].

Chromosome numbers were assigned based on the length of the aligned homologous chromosomes as displayed in GBrowse. Because most of the chromosome ends were not fully assembled (see below), the lengths of the chromosomes are likely underestimations. Compounding this is the fact that distinct, repetitive regions may be collapsed into a single gene or regions during assembly [23]. As such, the numbering scheme will not match those of previous studies [17-22,24]. In particular, chromosomes 1 and 3 as previously described [16,25,26] map to TcChr35 and TcChr6 respectively (see Discussion).

Figure 1 shows all 41 model chromosomes ordered by size, and Table 1 shows the breakdown of the genes/contigs/scaffolds included. Over 90% (21133 of 23216) of the genes annotated in the published genome are represented [3]. Tic marks under each chromosome show the location of gaps, indicating that the appropriate sequence for these regions was either not identified or that the sequence is not included in the released genome sequence [3]. The size of the chromosomes varies substantially (~78 kb to ~2.4 Mb) also in agreement with previous physical data [17-22,24]. These sizes are likely underestimates as not all contigs/scaffolds have been included because of the inability to confidently place many of the small contigs containing primarily members of the *trans*-sialidase, MASP, RHS, GP63, mucin, and DGF-1 familes, which collectively make-up approximately 23% of the annotated genes in the *T. cruzi *genome. In particular, the majority of the "unclosed" BAC clones (those for which the 5' and 3' ends were not oriented properly on the same chromosome) indicate that these sequences belong at the ends of the chromosome assemblies (telomeric and sub-telomeric regions). A total of 56 contigs containing telomeric repeats were identified. Of these, 39 were mapped to 22 of the chromosomes and the remaining 17 to the unassignable scaffolds and contigs. Of the 22 chromosomes containing telomeric repeats, 5 have these features on both ends (TcChr11, 22, 25, 27, and 35). Unassignable scaffolds and contigs are depicted as arbitrarily arranged assemblies in Figure 1.

**Table 1:** Breakdown of the genes/contigs/scaffolds on the assembled chromosomes

# Chromosomes (shortest: 78 kb, longest 2.3 Mb)	41
# Scaffolds on chromosomes	419

# Contigs on chromosomes	3338

# Genes on chromosomes	21133

# Unassignable Scaffolds	219

# Unassignable Annotated Contigs	663

# Unassignable Annotated Genes	2083

**Figure 1:**
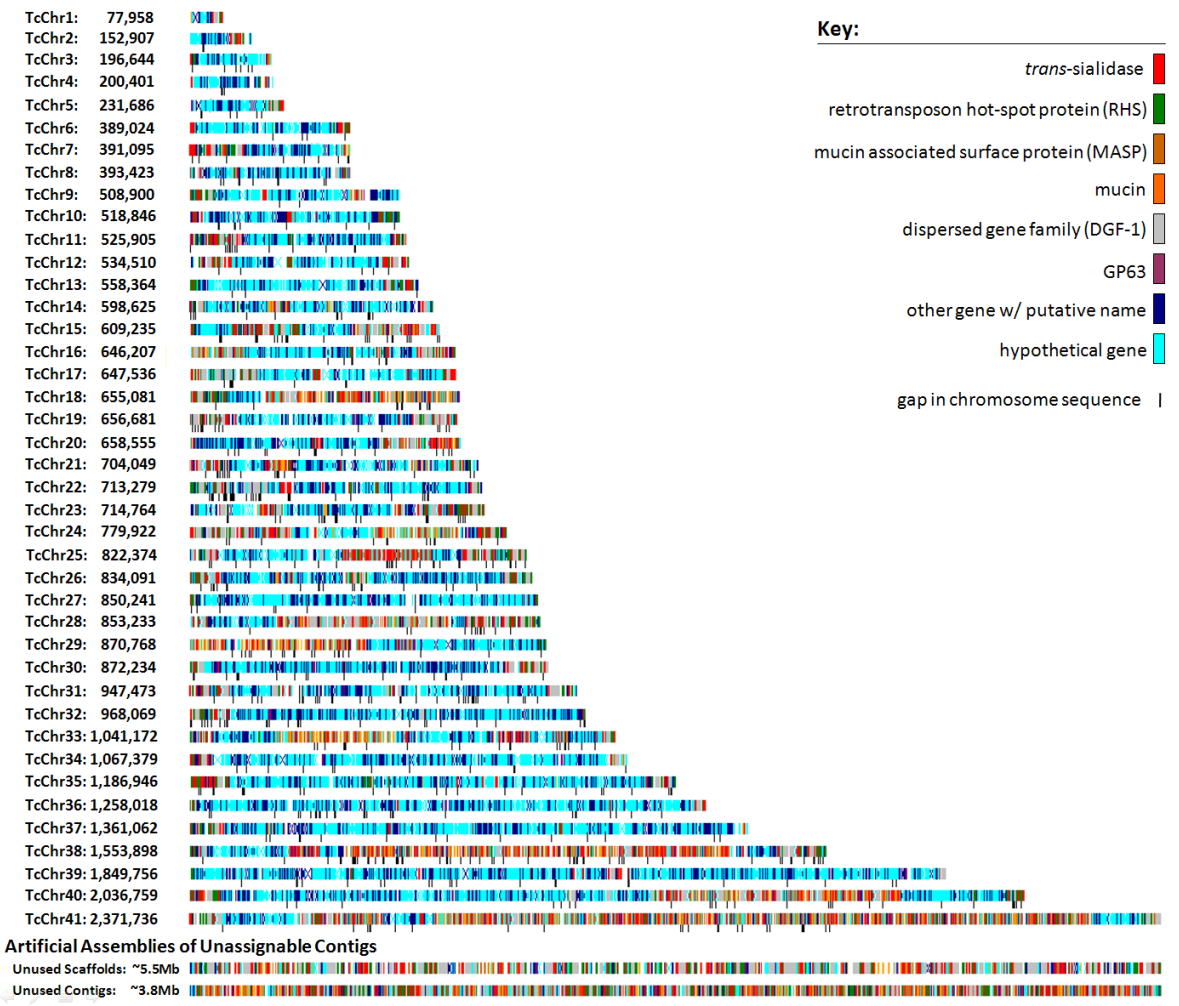
**The 41 model chromosomes of *T. cruzi***. Because the CL Brener reference strain is a hybrid of the "non-Esmeraldo-like" and "Esmeraldo-like" lineages, each chromosome is comprised of 2 homologous chromosomes. These model chromosomes represent the consensus view of both haplotypes. Gene family members are depicted as non-blue colors; of note is the number of clusters of gene family members on the chromosomes, as well as in the artificially assembled contigs that were not assignable to individual chromosomes.

### Visual Validation

**Figure 2:**
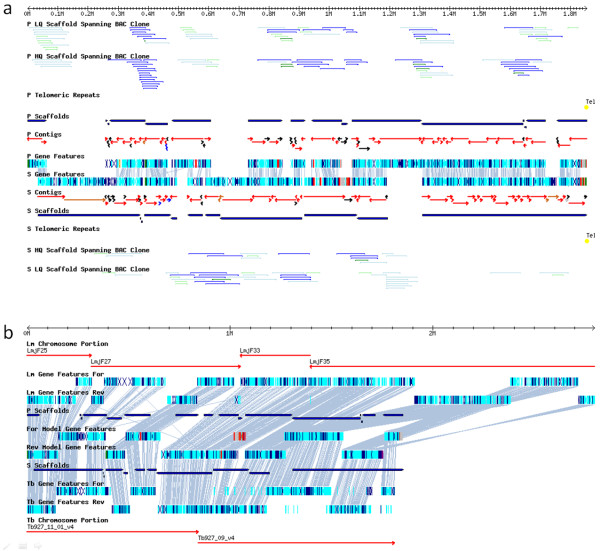
**Visual Validation**. a) The 1336 genes ("Gene Features"), 145 "Contigs", 26 "Scaffolds", and mapped BAC clones ("BAC Clone") are shown for the homologous chromosome pairs (the non-Esmeraldo-like haplotype are the "P" features and the Esmeraldo-like haplotype are the "S" features) of chromosome TcChr39. Where possible, contigs are aligned to place at least one pair of alleles in the same locus (red contigs). Lines between genes on the two homologous chromosomes indicate allelic synteny. BAC clones are color-coded according to the source library (blue = TARBAC, green = EPIFOS); note that only BAC clones that span scaffolds or whose ends map to opposite homologous chromosomes (light blue and light green) are shown. Black contigs are those that were not aligned with another on the opposite chromosome (either no homologous sequence present or the alignment could not be made). Finally, brown contigs indicate possible merged sequence from the two parental haplotypes, blue contigs denote sequences where one or more alleles exist on this chromosome but could not be aligned, and gray contigs indicate sequences where one or more alleles exist on a different chromosome altogether. b) The TriTryp synteny map of TcChr39 shows the regions of *T. brucei *("Tb Chromosome Portion") and *L. major *("Lm Chromosome Portion") for which at least 10 genes are syntenous with *T. cruzi *genes are shown. For each chromosome (i.e. Tc, Lm, Tb), the coding strand is shown ("Rev" for reverse strand and "For" for forward strand). Telomeric repeats were identified on the 3' end of this chromosome (yellow ellipses).

All 41 chromosomes were visually confirmed to ensure that sufficient evidence (allelic synteny, mapped BAC clones linking scaffolds) exists to support the organization. For example, Figure 2a shows a view of chromosome 39 (TcChr39) where only the BAC clones that either span scaffolds or whose ends map to homologous regions on the paired chromosomes (and thus indicative of missing sequences on one of the chromosome pairs – see Discussion) are displayed. This chromosome is comprised of 145 contigs and 16 scaffolds, five of which were split in order to place genes on the appropriate chromosome of the homologous pair. The cumulative mapping of multiple BAC clones linking all scaffolds is strong support for the accuracy of the chromosome organization. As expected, the *T. cruzi *chromosome exhibits substantial synteny with regions of the chromosomes of *T. brucei *as well as those of *L. major *(Figure 2b).

### Experimental Validation

**Figure 3:**
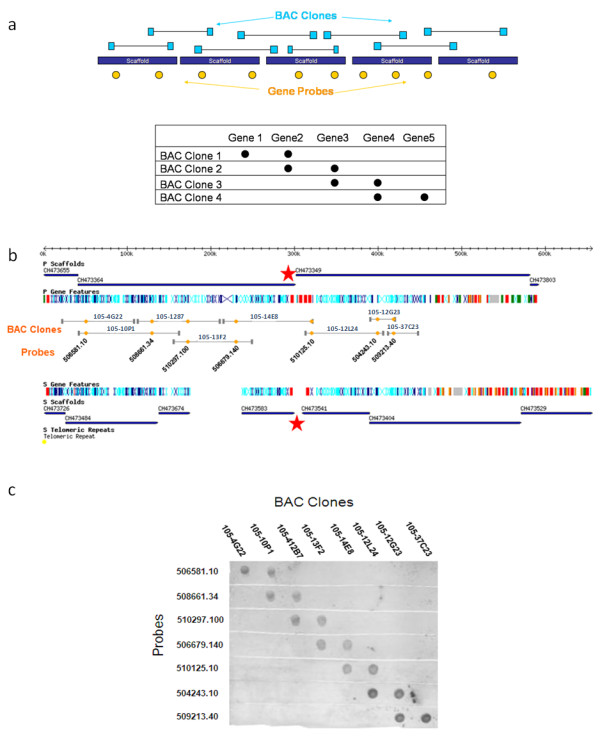
**Validation of assemblies by Southern "dot" blot analysis**. a) The top-most cartoon depicts overlapping BAC clones (light blue) which are predicted to span a chromosome (assembled from multiple scaffolds, dark blue). Genes (orange) are selected such that they link proposed contiguous scaffolds. These overlapping pairs of probes create a stair-stepping effect on the Southern blot if the hybridization results are as predicted (bottom), indicating that the linkage of the scaffolds, and thus the chromosome, is correct. b) TcChr20 was chosen for Southern blot validation. Overlapping BAC clones (grey) are selected such that they span most of the chromosome. Gene probes are shown in orange (ids shown are truncated forms of the "Tc" gene ids, i.e. 506581.10 denotes Tc00.1047053506851.10). While there are 11 previously published scaffolds linked on this chromosome, the red stars indicate a region containing many *trans-*sialidase genes whose repetitive nature must have hindered the joining of the 4 scaffolds that terminate in this region (2 large "P" scaffolds and 2 "S" scaffolds). c) For each BAC clone, both of the gene probes positively hybridize as predicted, creating the stair-step effect that confirms the linkage of the genes on the chromosome. BAC clones are labeled with their assigned ids from the CHORI library.

We initially sought to validate the linkage of genes on the same chromosomes using data from previously published physical maps [16-18,20-22,27]. However, this effort was ultimately inconclusive, as too few of the described markers mapped unambiguously to single chromosomes. Rather than attempt further chromosomal blot analyses, we chose to validate the method of chromosome assembly by probing mapped, overlapping BAC clones with specific genes that the clones were predicted to contain based upon our assembly (Figure 3a, b). Figure 3c shows that in all cases, the probes hybridized with the expected BAC clones. These results substantiate the utility of our methods, as the linkage of 2 large scaffolds on the non-Esmeraldo-like "P" chromosome and 5 scaffolds on the Esmeraldo-like "S" chromosome was confirmed. The gap in the middle of the chromosome between scaffolds CH473364 and CH473349 on the P chromosome and CH473583 and CH473541 on the S chromosome (denoted by red stars on Figure 3b) shows a region rich in *trans*-sialidase sequences that may have hindered the linkage of these scaffolds in the original assembly. Although the complete chromosomal assembly was not validated by dot blot analysis, similar probing of other selected chromosome regions also confirmed the quality of the assembly (Additional File 1 and text below).

### Additional "fixes" in the reassembled *T. cruzi *genome

Although in most cases we accepted previously assembled scaffolds as correct, there were occasions where this approach was not compatible with synteny and BAC-end mapping results. In most of these cases, sets of homologous genes from the 2 distinct haplotypes in the CL Brener genome had been placed adjacent to each other in the formation of scaffolds and contigs. To correct for this issue, scaffolds were split (always at contig boundaries) in order to assign genes to the appropriate homologous chromosome (Additional file 2). For example, on TcChr39 scaffold CH473328 was divided into 2 pieces and these pieces assigned to the P and S chromosomes in order to place the respective alleles on the appropriate chromosome. This assignment is consistent with the annotation of these alleles as heterozygous and as part of the same COG group [3]. Overall, 166 scaffolds were split to correct for the presumed merging of homologous sequences from the 2 haplotypes.

TcChr39 illustrates a second type of sequence merging: that of homologous Esmeraldo- and non-Esmeraldo-like raw sequence reads during the creation of the consensus sequence (Additional file 2), the evidence for which is the fact that the "best hit" mappings of BAC ends of the same clone were to contigs assigned to different chromosomes. For example, the 5' BAC end of clone "CHORI105-17N2" maps to the P chromosome on contig 504109 with 99.9% sequence identity (840/841 bases), while the 3' end maps to the S chromosome on contig 506925 with many mismatches (97.2%, 714/734 bases). Additionally, 4 genes on contig 506925 are annotated as "heterozygous" but have their predicted allelic match on the same contig. Errors of this type are noted where observed but the reassembling of these merged contigs throughout the genome was considered to be beyond the scope of this work.

### Observations from the Assembled Chromosomes

**Figure 4:**
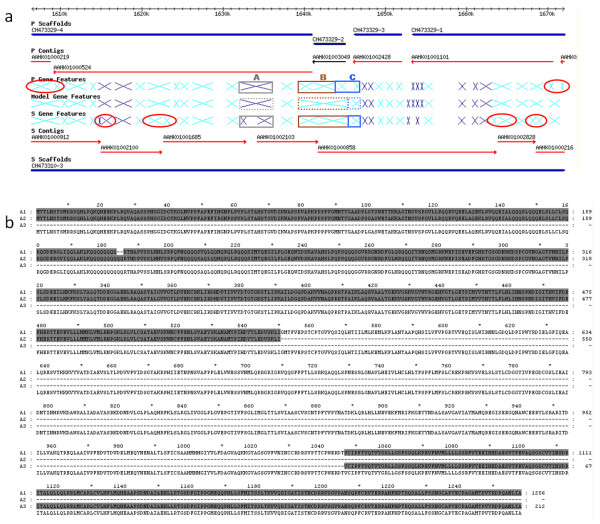
**Differentiation of alleles from paralogues**. a) The solid boxes highlight assembly issues with the current genome. The solid gray boxes (locus "A") show a *helicase *gene where 1 allele is fully sequenced on the P chromosome and the other is in 2 pieces on S. The solid brown boxes (locus "B") show a *hypothetical protein *with no fully sequenced copy: the 2 genes on the P chromosome and 2 genes on the S chromosome both reside at contig boundaries and should be merged. Finally, the solid blue boxes (locus "C") show a case of 2 copies (at least partially sequenced) on the P chromosome and 1 presumed fully sequenced copy on S of the same *hypothetical protein*. In all cases, the dotted line boxes indicate the predicted correct coding sequences. Note that the circled genes show other cases of gene truncations at contig boundaries. b) Alignment of 3 annotated genes in the A locus (a *helicase *gene). The A1 allele is full length, while A2 and A3 are small pieces which exist on the ends of contigs and are not fully sequenced. Note that A2 has near perfect identity with the N-terminus of A1, while A3 has perfect identity with the C-terminus suggesting that these are pieces of the same gene.

Figure 4 illustrates how the assembled *T. cruzi *chromosomes can help clarify gene assembly issues. In the region of TcChr39 (Figure 4a) the genes flanking those labeled A through C show strong synteny between the assembled P and S chromosomes. However, genes A and C each have one copy on one of the homologous chromosomes but two annotated genes on the corresponding chromosome, while B has 2 differently sized copies on each chromosome. In all cases, it appears that the "2 copies" are actually truncated pieces of the full-length gene, as shown by sequence alignment for gene A, which encodes a helicase protein (Figure 4b). These truncations all occur at contig boundaries – and indeed there are at least 6 additional sets of truncations apparent in this one section of chromosome. Careful comparisons of the assembled chromosomes should allow for many of these events to be corrected.

**Figure 5:**
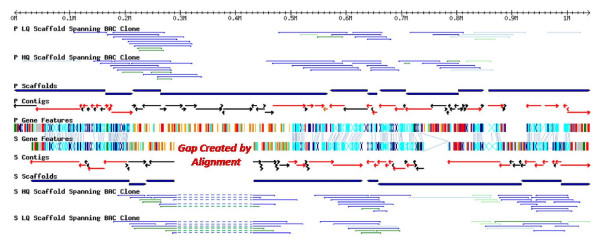
**Cluster of large gene family members in the middle of TcChr33**. The region from ~0.2 Mb to 0.5 Mb contains mostly gene family members (non-blue "Gene Features"), while on either side of the region are "core" regions with either hypothetical genes or those with an assigned putative function (light and dark blue "Gene Features"). The large number of spanning BAC clones linking the core regions and the cluster of gene family members substantiates the organization. However, it should be noted that these homologous chromosomes are likely different sizes. The BAC clones on the "S" chromosome that span the 120 kb gap in the gene family rich region (connected by dashed lines) are too long for the BAC libraries as shown (TARBAC: blue, avg. length 75 kb, EPIFOS: green, avg. length 35 kb). Alignment of the homologous chromosomes is a visual aid to maintain allelic synteny only.

One of the unique aspects of the *T. cruzi *genome among most sequenced genomes is that it contains at least 22 separate gene families with 20 to >1400 members [3]. Because of the sequence similarity between members of these large gene families, it was not possible to unambiguously map many of the contigs rich with gene family members, leaving them to be included among the "artificial" assemblies in Figure 1. However, of the gene-family-rich contigs that are included in the assembled chromosomes, many cluster at chromosome ends while others are located in the middle of chromosomes (13 clusters of 10 or more members and 6 clusters of > = 20 members that are flanked by regions containing > 10 core genes; Figures 1, 5). Figure 5 also demonstrates that size variation of chromosomes in *T. cruzi *is due not only to variation at chromosome ends but also variation in the composition of chromosome-internal sites rich in gene family members.

**Figure 6:**
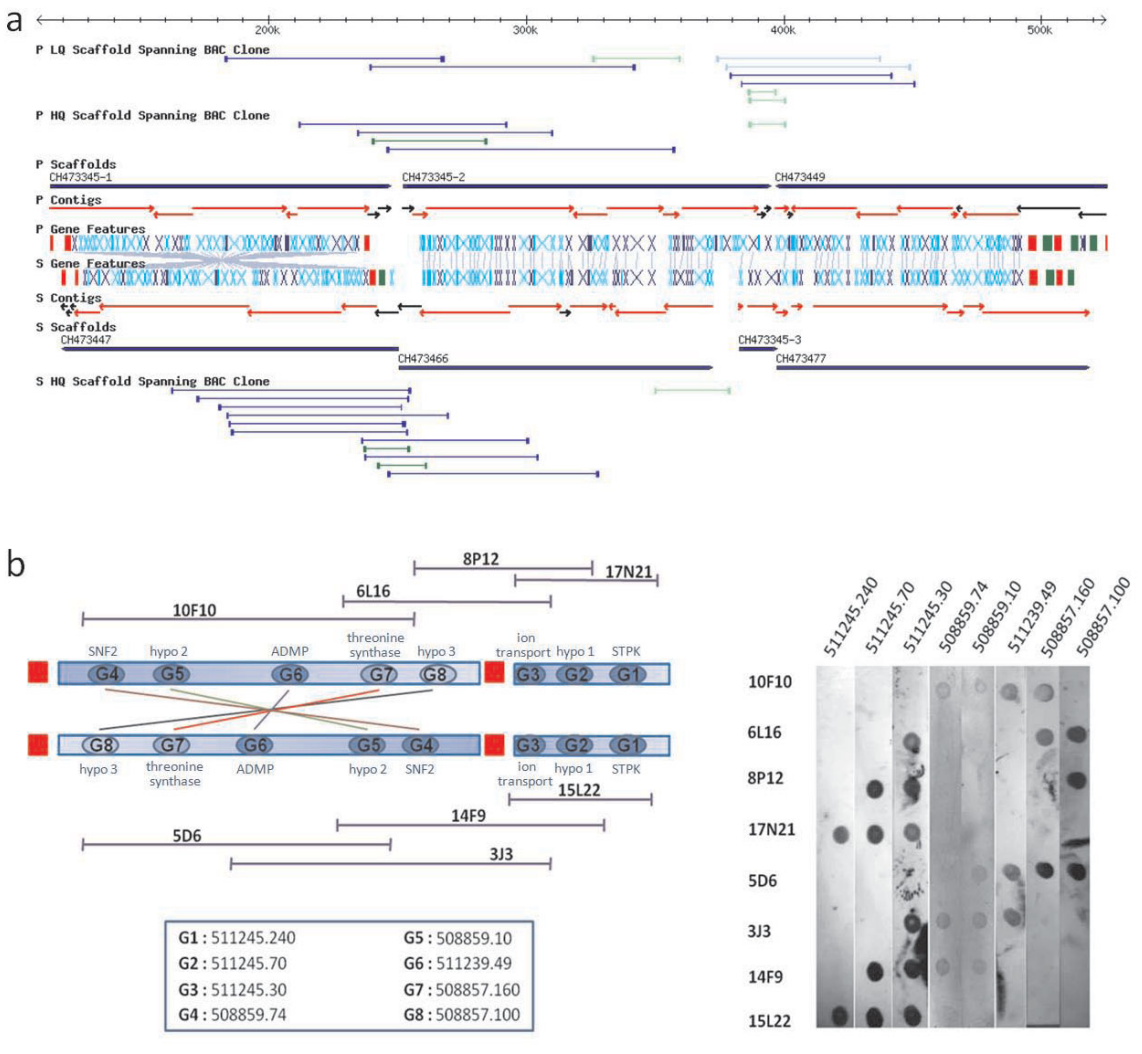
**Local inversion on *T. cruzi *TcChr11**. a) The region on left-hand side of homologous chromosome P appears inverted relative to S, as the orientation of scaffold CH473345-1 was reversed relative to CH473447 in order to close the BAC clones on both homologous chromosomes (i.e if CH473345-1 was oriented according to allelic synteny, then the BAC clones on the P chromosome would be unclosed). b) BAC clones and probes were chosen for Southern blot analysis to validate this inversion. The left-hand side shows a diagram of the design of the blot (see Figure 4 for more details). The chromosome inversion is confirmed if BAC clones 15L22, 14F9, 3J3, and 5D6 contains genes G1–G7 (but not G8), but BAC clones 17N21, 8P12, 6L16, and 10F10 contain genes G1–G3 and G5–G8 (but not G4). The right-hand side of the figure shows the result of the analysis where the hybridization results confirm the inversion.

Lastly, the *T. cruzi*-based TriTryp synteny maps show many examples of apparent chromosomal rearrangements that have occurred since *T. cruzi*, *T. brucei*, and *L. major *diverged. In several cases, large pieces of otherwise syntenous regions of chromosomes show reversed orientation, typically near strand-switch regions. One particularly interesting chromosome is TcChr11, where there appears to have been a local inversion of a ~125 kb piece within one of the homologous chromosomes (Figure 6a). It is only with the reversal of scaffold CH473345-1 on the S chromosome, in relation to the homologous region on the P chromosome, that the BAC clones mapping to each chromosome can be oriented appropriately. Dot blot analysis validates this orientation (Figure 6b).

## Discussion

In this work we have attempted to complete the job of assembly of the *T. cruzi *CL Brener genome using all available sequence information. The result is a genome of 41 chromosome pairs ranging in size from 78 kb to 2.4 Mb. This assembly is somewhat unique to currently sequenced genomes in that both homologous chromosomes of this widely heterozygous hybrid strain required construction before a consensus model for each chromosome could be derived. Previous pulsed field gradient electrophoresis (PFGE) studies [17-22] have estimated that the chromosomes of *T. cruzi *range in size from 300 kb to over 3 Mb. While the range of the assembled chromosomes is less than those from the PFGE studies, the difference is likely due to the gene family rich contigs that were not able to be placed in this assembly. However, a previous orthogonal-field-alternation gel electrophoresis (OFAGE) study [24] has described chromosomes as small as 100 kb.

Previous chromosome-level studies [16,25,26] in *T. cruzi *have focused on two assemblies named chromosomes "1" (corresponding to TcChr35 herein) and "3" (TcChr6) based on the ordering of gel bands in PFGE analyses. According to these studies, chromosome "1" exists as 2 homologous chromosomes of size 450 kb and 1.3 Mb, while chromosome "3" is present as 2 homologs of 600 kb and 1.0 Mb. However, while the PFGE analyses predict different sized homologous chromosomes for both "1" and "3", in our assemblies the homologous chromosomes of each are roughly the same size (> 1 Mb for TcChr35 and ~400 kb for TcChr6). In the case of chromosome "3" (TcChr6), where the size of the assembled homologous chromosomes is smaller than their estimated lengths from the PFGE study, it is likely that the size discrepancy is due to unassigned gene family rich contigs in the sub-telomeric and/or telomeric regions. This justification is consistent with the previous finding that the deletion of the 400 kb sequence responsible for the size differences of the homologous chromosomes of "3" resulted in no phenotypic consequences [16]. However, there is a clear contradiction between the organization of TcChr35 and the model for chromosome "1" proposed in [16] as both assembled homologous chromosomes in TcChr35 are larger than the reported 450 kb homolog. This study notes that the difference in size between the two homologs of chromosome "1" cannot be due to additional sequence between the *Tcsod *locus and the downstream telomere. However in the assembled TcChr35, the *Tcsod *locus is essentially in the middle of the homologous chromosomes. Synteny between the chromosomes of *L. major *(Lm Chr32) and *T. brucei *(Tb Chr11) supports the organization of TcChr35 as assembled herein, as does the BAC clone mappings of the Esmeraldo-like homologous chromosome. However, despite the allelic synteny across the entire chromosome, the lack of spanning BAC clones on the non-Esmeraldo-like chromosome from the *Tcsod *locus to the rest of the chromosome does not rule out the possibility that there exists a small homolog of that chromosome ending near the *Tcsod *locus (as described for chromosome "1" in [16]). If this were the case, then the remainder of the non-Esmeraldo-like chromosome is either a separate chromosome homolog or is a portion of another chromosome altogether.

The fixes and observations described above emphasize perhaps the most confounding issue with using the initial *T. cruzi *genome assembly [3]. Copies of heterozygous alleles are often annotated as independent genes when in fact they are alleles on the homologous chromosomes in this hybrid strain (a problem with the assembly). At the same time, many families of genes include truly distinct genes in discrete loci (an aspect of the organism). These characteristics make it challenging to determine when genes are heterozygous alleles mapping to the same locus or are paralogous genes at different loci. This latter decision is of course further complicated by the fact that the complete sequence for many genes is not present in the assembled contigs. Viewing regions of the assembled chromosomes where both haplotypes are represented facilitates this determination; special consideration must be made for syntenous genes where one or both of the alleles exist at the end of contigs because many of these are truncated and should be merged with another "gene" on the adjacent contig.

The *T. cruzi *genome contains many non-gene-family, homozygous genes (i.e. with only a single annotated allele) that disrupt the allelic synteny of homologous chromosomes (Figure 2a, Additional file 2). These sequences are likely the result of the merging of sequence from both the Esmeraldo- and non-Esemeraldo-like haplotypes, an indication that the homologous chromosomes are, as expected, quite similar. However, there are many sequenced BAC ends whose exact sequence does not exist in the annotated genome, such as in cases where one BAC end maps to a contig on a particular homologous chromosome with near perfect sequence identity, while the best match of the other end is to a contig on the other homologous chromosome with many mismatches (Additional file 2). Given that the genome was sequenced to 14× coverage at an error rate of < = 1.5%, the absence of these sequences is surprising. Further examination of the raw sequence reads may reveal that the particular sequences exist but were not utilized in the assembly process. Regardless, the current analysis has mapped to these chromosomes the candidate BAC clones that could be fully sequenced in order to correct these errors and close the remaining gaps in the chromosomes.

The assembled chromosomes provide a physical platform on which to study gene function and variation in *T. cruzi*. For example, the chromosome structure provided here will be particularly useful for planning and confirming gene knockouts and thus determining the function of hypothetical genes or confirming the function of annotated genes. As RNAi does not appear to function in *T. cruzi*, gene knockout remains a primary method linking phenotypes or functions to particular gene products. In addition, the chromosomes will facilitate strain comparisons, either by techniques such as CGH or subsequent sequencing of additional strains.

Telomeric and sub-telomeric regions of the chromosomes may never be fully sequenced; these regions are likely too redundant to assemble properly and yet too variable as a whole between strains of *T. cruzi *to be ultimately informative, except as examples of the degree of variability that is possible in *T. cruzi*. As assembled, over 23% of the annotated genes in the genome are members of large gene families, but it has been suggested that there may be upwards of 20,000 additional genes in these families that are not present in the genome due to the collapsing of reads during assembly [23]. The large number of gene families and the substantial number of members of these families will be interesting to further explore, as the biological function of such large and diverse families of genes is not totally clear. It is hypothesized that the location near chromosome ends facilitates rearrangement in these genes and thus provides a source for new variants [28]. Since members of these families are major targets of anti-*T. cruzi *immune responses, it is likely that this variation has a role in immune evasion. It would be of interest to determine if gene family clusters that are integrated amongst the core genes in the *T. cruzi *genome are less prone to rearrangement over time or variation between strains relative to those on chromosome ends, as would be predicted.

As a caveat, one of the risks in assembling the chromosomes as described here is that a mosaic may result given the repetitive and hybrid nature of the *T. cruzi *genome. Though the majority of BAC clones used for organization were mapped unambiguously to the appropriate chromosomes, it must be noted that the organization was based on the most likely location of each scaffold/contig; there were many clones whose BAC ends either mapped to different chromosomes, a contradiction to the placement of the associated sequences, or mapped to scaffolds/contigs that were not placed on any chromosome. Thus these assemblies represent a model for the chromosomes of *T. cruzi*, and, though they are still incomplete, they are a vast improvement on what was previously available.

## Conclusion

The *T.cruzi *genome that was published in 2005 provided a foundation for discovery-based research of this human pathogen. However, because of the repetitive nature of the genome and the fact that the reference strain is a widely heterozygous hybrid of two distinct lineages, sequencing and assembly was hindered and resulted in the publication of many small pieces instead of complete chromosomes, as was the case for *T. brucei *and *L. major*. In this work, the small pieces (contigs and scaffolds) were assembled into chromosomes based on the predicted parental haplotype, inferences from the TriTryp synteny maps, and end sequences from *T. cruzi *BAC libraries. The approach was substantiated through the use of Southern blot analysis to confirm the mapping of the BAC clones using as probes the genes they are predicted to contain, and each chromosome was visually validated to ensure sufficient evidence (BAC clones linking scaffolds) was present to support the organization. These chromosomes extend the foundation of the published genome to facilitate gene-level analyses, such as gene knockouts, by assisting the determination of the number of copies of a particular gene and global analyses such as CGH studies for comparisons of chromosomal differences between strains of *T. cruzi*. The assembled chromosomes, as well as the mapped BAC clones that were used to construct them, are available on TriTrypDB, a trypanosome database of the EuPathDB organization [29].

## Authors' contributions

DBW assembled and validated the chromosomes and drafted the manuscript. CB performed the dot blot validations. RLT is principal investigator. He provided critical input and feedback on all phases of the project and assisted in manuscript preparation. All authors have read and approved the final manuscript.

## Supplementary Material

Additional file 1 **Dot-blot validation of a large region of TcChr39**. The organization of a 1.4 Mb region was validated using the described dot blot methodology. The file consists of 5 figures (a-e) and a legend. Click here for file

Additional file 2 **Merging of sequences from both haplotypes and splitting of scaffolds**. Some scaffolds from the TSKTSC ver5 genome contained sequences mapping to both parental haplotypes of the CL Brener reference strain and were split accordingly. The file consists of 2 figures (a, b) and a legend. Click here for file
