# Supplementary material for: Chromosome level assembly of the hybrid Trypanosoma cruzi genome
Source: BMC Genomics. 2009 Jun 1;10:255. doi: 10.1186/1471-2164-10-255 (PMC2698008; doi:10.1186/1471-2164-10-255)
Supplement: Additional file 1 — Dot-blot validation of a large region of TcChr39. The organization of a 1.4 Mb region was validated using the described dot blot methodology. The file consists of 5 figures (a-e) and a legend. [file 1471-2164-10-255-S1.doc]

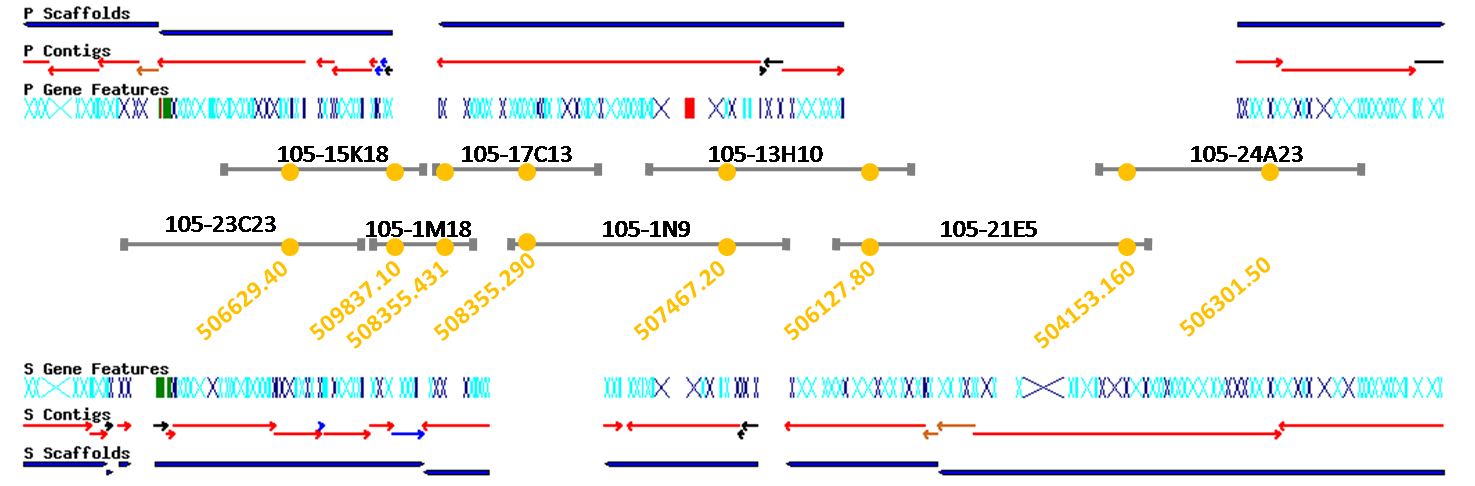


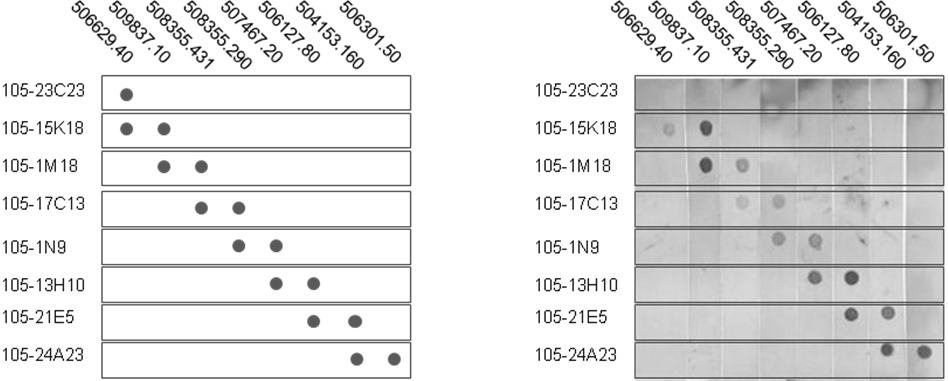


Supplemental Figure 1a


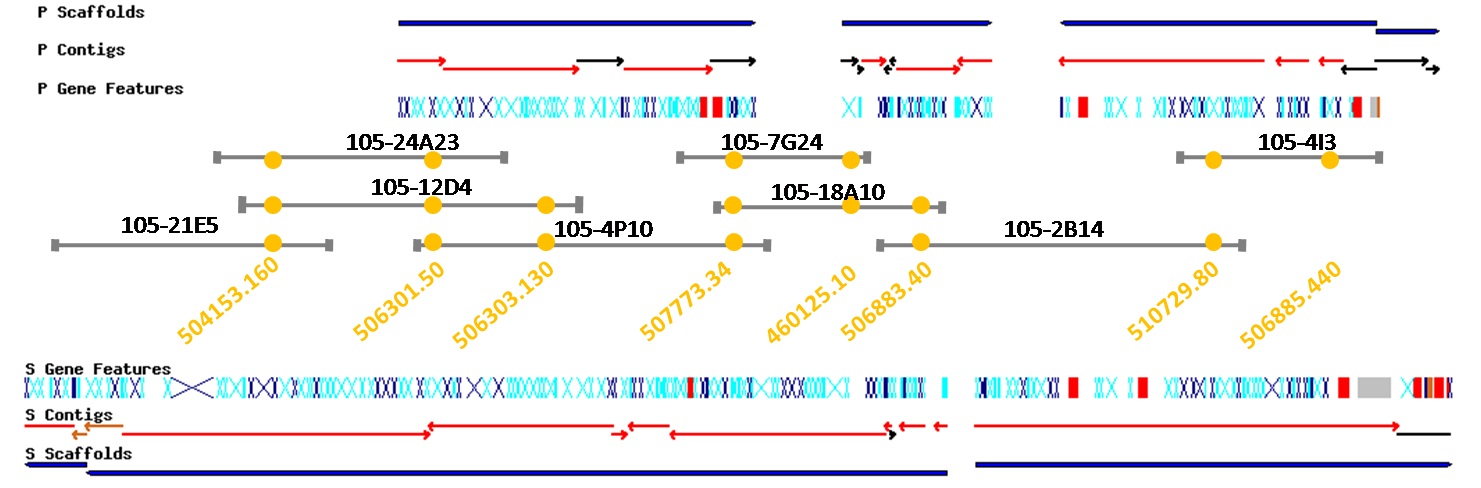


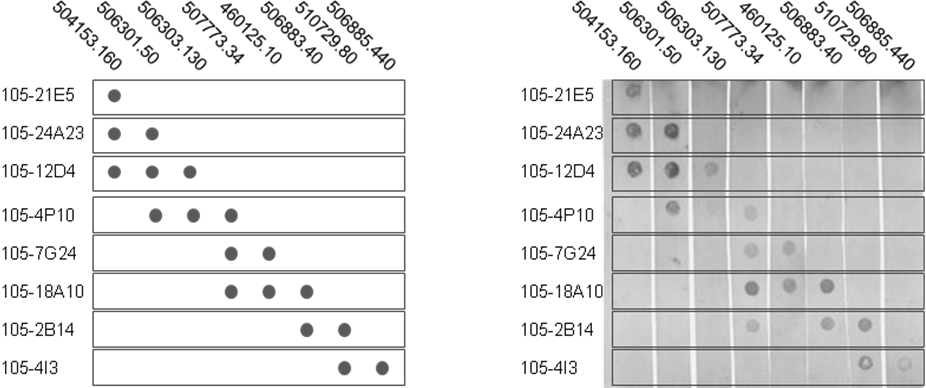


Supplemental Figure 1b


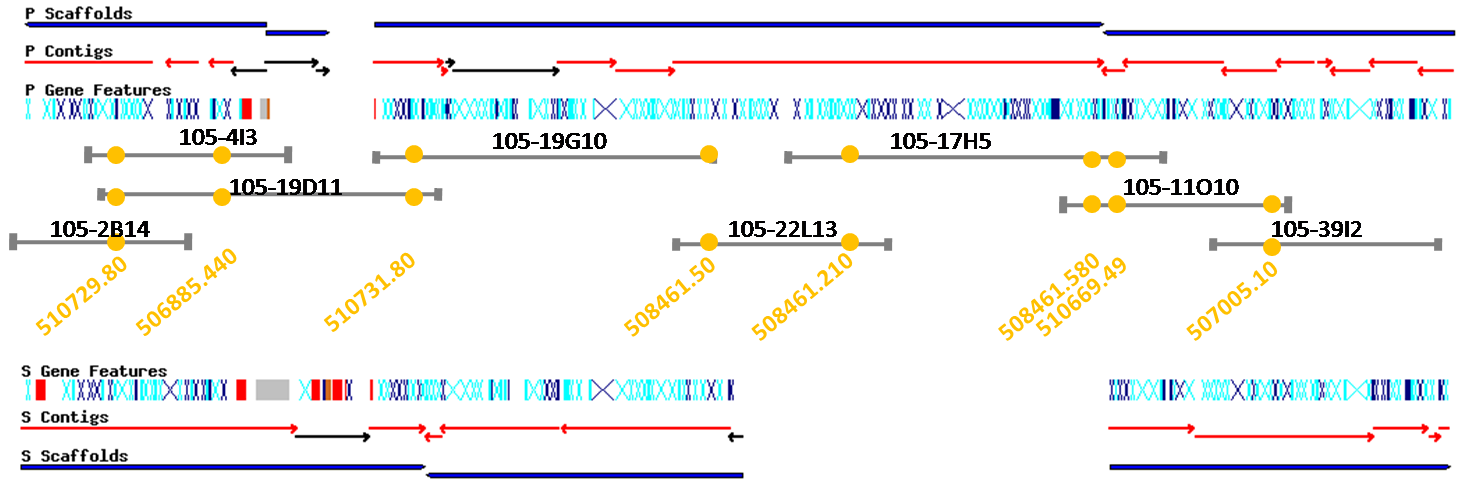


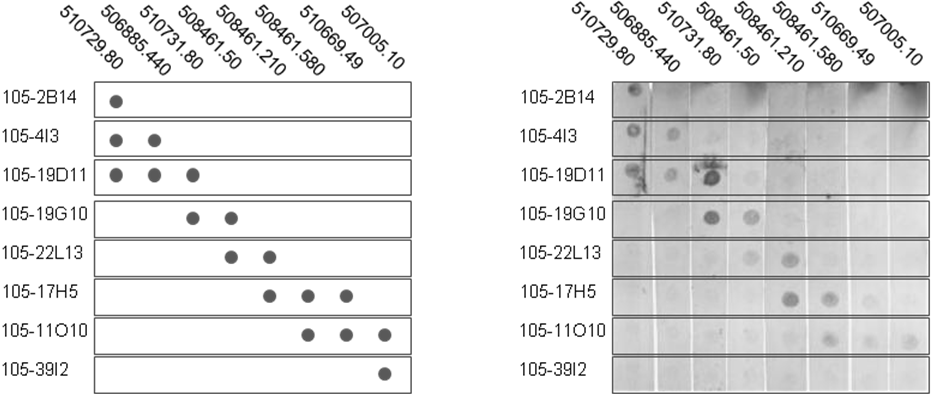


Supplemental Figure 1c


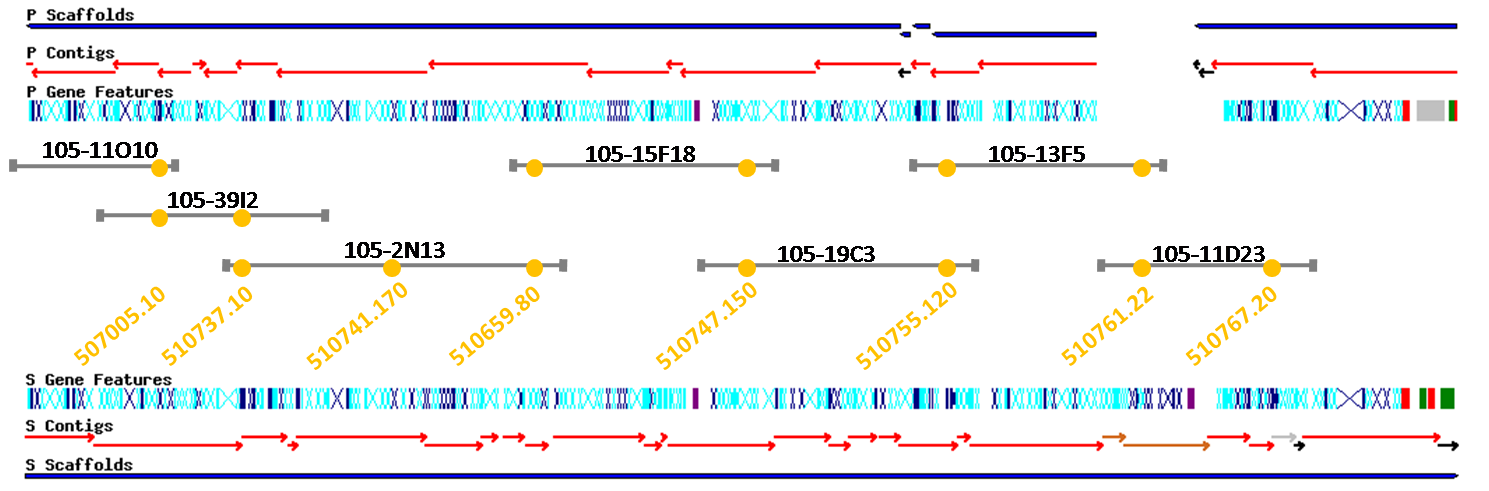


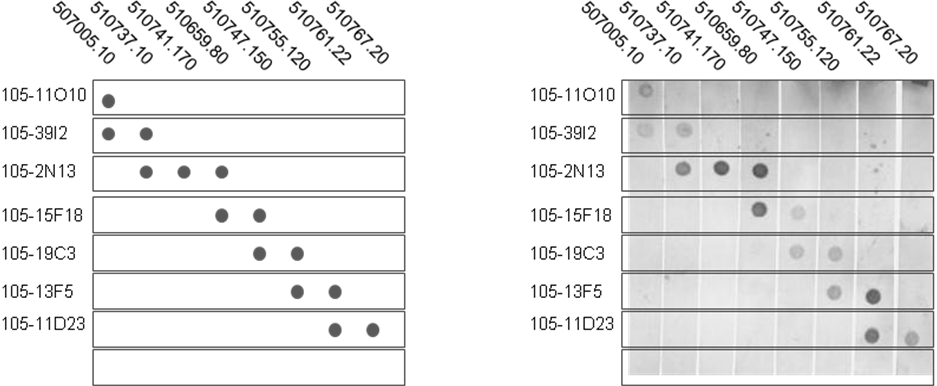


Supplemental Figure 1d

Supplemental Figure 1: Dot blot Validation of a large region TcChr39. a-d) 4 dot blots were performed for different sections of TcChr39 that span 1.4Mb of the chromosome (see Figure 2b and 3 of the manuscript for view of whole chromosome and more detail about dot blot analysis). Note that the last 1-2 rows of a dot blot are the first rows of the next dot blot (105-11O10 and 105-39I2 are the last rows of d and the first rows of e). The blots confirm the organization of this region of the chromosome (105-39I2 is negative in c but positive for the same probes in d).
