# Supplementary material for: Chromosome level assembly of the hybrid Trypanosoma cruzi genome
Source: BMC Genomics. 2009 Jun 1;10:255. doi: 10.1186/1471-2164-10-255 (PMC2698008; doi:10.1186/1471-2164-10-255)
Supplement: Additional file 2 — Merging of sequences from both haplotypes and splitting of scaffolds. Some scaffolds from the TSKTSC ver5 genome contained sequences mapping to both parental haplotypes of the CL Brener reference strain and were split accordingly. The file consists of 2 figures (a, b) and a legend. [file 1471-2164-10-255-S2.doc]

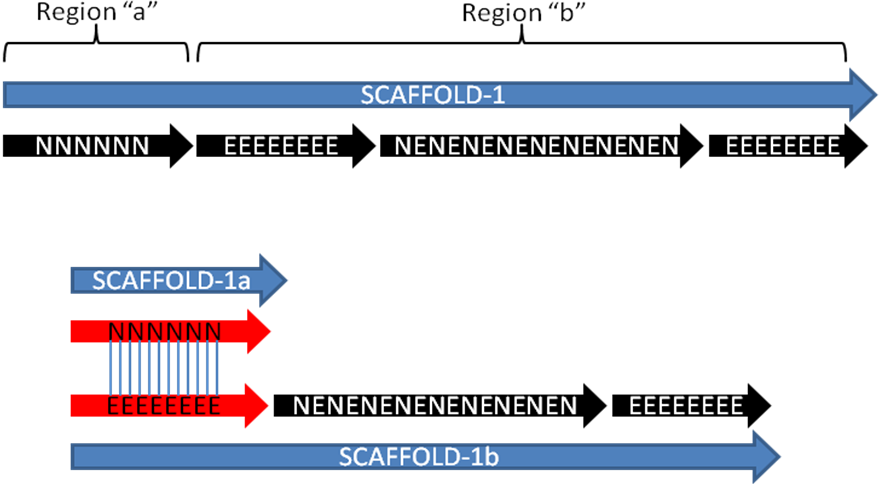


Supplemental Figure 2a


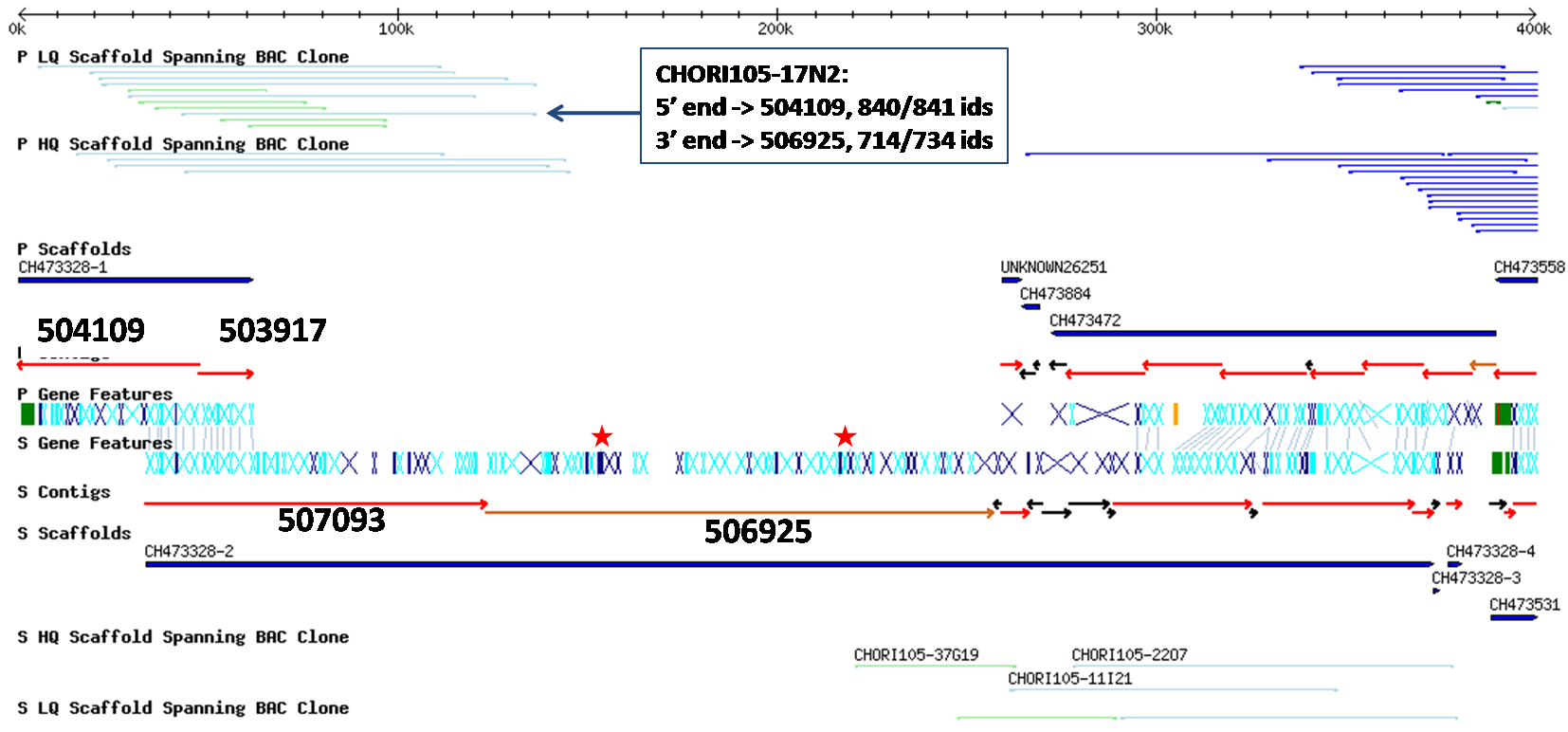


Supplemental Figure 2b:

Supplemental Figure 2: Merging of sequences from both haplotypes and splitting of scaffolds. a) Some scaffolds contained sequences mapping to both haplotypes: contigs consisting of “E”s represent the Esmeraldo haplotype, while contigs consisting of “N”s represent the non-Esmeraldo haplotype. Contigs resulting from merging of sequence during their assembly contain both Ns and Es. In cases where entire contigs could be assigned to a particular haplotypes, the scaffolds were split to map the contigs to the appropriate homologous chromosome. However, in cases where the sequence for an individual contig was the result of the merging of sequence during its assembly, no splitting was performed. b) Genes on scaffold CH473328-1 (contigs 504109 and 503917) are annotated as belonging to the non-Esmeraldo-like haplotype, and the syntenous genes on scaffold CH473328-2 are annotated as the Esmeraldo-like haplotype (note that the contig ids are abbreviated, e.g. 504109 is Tc00.1047053504109). To place the genes on the appropriate homologous chromosome, the scaffold was split to put CH473328-1 on the non-Esmeraldo-like “P” chromosome. Conversely, most of the genes on contig 506925 are annotated as being the result of the merging of sequences from both haplotypes. This merging is further highlighted by the light-blue and light-green BAC clones which indicate the lack of specific sequence assignable to a particular haplotype, as the ends for the same BAC clones map to different homologous chromosomes. Interestingly, 4 of the genes on this contig are annotated as heterozygous with both genes (likely alleles) present on the contig (the red stars indicate the position of pairs of adjacent genes), but splitting of contigs was not performed to resolve these.
